# On the Reality of Intrinsically Finkable Dispositions

**DOI:** 10.1007/s11406-016-9713-z

**Published:** 2016-04-30

**Authors:** Matthew Tugby

**Affiliations:** Department of Philosophy, Durham University, Office 004, 51 Old Elvet, Durham, DH1 3HN UK

**Keywords:** Dispositions, Intrinsicality, Finks, Explanation, Negative properties

## Abstract

Recently, Choi (*Noûs, 46*, 289–325, 2012) has argued that current accounts of intrinsically finkable dispositions lead to absurd consequences in certain everyday cases. In this paper I offer a new argument for the existence of intrinsically finkable dispositions, one which provides a new way of testing for the presence of such dispositions. It is then argued that, with this new test in place, Choi’s examples no longer present a problem for the view that some dispositions are intrinsically finkable.

## Introduction

In contemporary metaphysics, few deny that dispositions can truly be ascribed to things. But there are still cases in which it is unclear as to whether a thing has a certain disposition. For example, it has been questioned whether there can be intrinsically finkable dispositions (the notion of finkablity will be explained below). And as Choi points out (Choi 2012, p. 289), this is an important question, as it affects issues in a variety of philosophical topics. On one side of the debate are those who favour analyses of dispositions which rule out the possibility of intrinsically finkable dispositions (e.g. Choi 2005, 2012; Handfield 2008 and Handfield and Bird 2008). In contrast others have happily accepted that there can be intrinsically finkable dispositions (e.g. Clarke 2008, 2010 and Fara 2008).

In a recent paper, Choi (2012) has defended his rejection of intrinsically finkable dispositions (henceforth ‘IF dispositions’) against the opposing views of Clarke and Fara.^1^ After showing that his analysis of dispositions entails that IF dispositions are impossible, Choi argues that the sort of reasoning that leads Clarke and others to accept the possibility of IF dispositions will lead them to ascribe absurd dispositions in certain everyday cases. I largely agree with Choi’s assessment. But all is not lost for the defender of IF dispositions. In this paper I offer new reasons for thinking there can be IF dispositions, reasons which suggest a new criterion for identifying such dispositions.^2^ I then argue that this new criterion delivers the correct verdicts in Choi’s problem cases. But before presenting the core arguments, we must first explain in more detail the notion of finkability.

## Finkable Dispositions

The notion of a fink was first introduced by C.B. Martin (1994, p.2) and refers to a factor which causes a disposition to be lost as soon as it is triggered, thereby preventing the disposition’s manifestation from coming about. Martin’s original example of a fink, known as the electro-fink case, is one in which the finking factor is *extrinsic* to the object whose disposition is frustrated. In the 'reverse' electro-fink case (Martin 1994, p.3), the fink is an external machine that detects when a live wire comes into contact with a conductor. The instant at which such contact occurs, the fink causes the wire to become dead, so that the flow of electricity is prevented. Here, then, we have a case in which the wire plausibly has a certain disposition even though the associated manifestation is prevented by an extrinsic interferer.

Now, given that metaphysicians have invariably accepted that there can be extrinsically finkable dispositions, one would naturally assume we should accept cases in which the finking factor happens to be intrinsic to the thing with that disposition. And indeed, people like Clarke (2008) and Fara (2008) have happily accepted the possibility of IF dispositions. In reconstructing the sort of reasoning that people like Clarke employ, Choi (2012, pp. 305-306) explains that the acceptance of IF dispositions is typically driven by two observations. The first observation is that in alleged cases of IF dispositions, the object in question has a part that we would typically agree confers the disposition in question. For instance, although someone may fail to lift heavy things due to a certain intrinsic property, if they had the same muscular structure as people who are clearly strong, this could provide a reason for thinking the person in question is also strong (Clarke 2008, p. 513). The second observation is that because intrinsic finks play the same kinds of causal roles as extrinsic finks, and we are all happy to accept the existence of extrinsically finkable dispositions, we should therefore accept, for reasons of parity, that there can be IF dispositions.

But as indicated above, the problem is that, as Choi shows, this sort of reasoning arguably leads to the ascription of absurd dispositions in certain everyday cases. Does this mean that the reasoning just outlined is seriously mistaken? I do not think so. Both reasons above are an important part of the story about why we should be willing to ascribe IF dispositions in certain cases. But it is not the whole story. In the next section, I will provide a new argument in favour of IF dispositions, one which suggests a new way of testing for the presence of an intrinsically finkable disposition.

## In Support of intrinsically Finkable Dispositions

The imaginary but physically possible case that I will use for illustrative purposes is as follows. Imagine a robot that has enough electrical charge running through its body to kill any living being that touches it, as long as its battery remains connected upon contact. Imagine further, however, that at any moment at which the robot is touched by a living being, the battery is automatically disconnected by the robot’s charge modulator. Because of the robot’s modulator, it is safe for people to touch the robot even though it may seem natural (given the presence of the charged battery) to suppose that the robot has the disposition to electrocute. If this natural supposition is correct, then this is a case of a disposition which is susceptible to an intrinsic fink. I will call this robot ‘Batfink’.

It is, I suggest, problematic to deny that Batfink has the disposition to electrocute. An initial cause of unease, of a kind that has been highlighted by Ashwell (2010, p. 649), is that the denial appears to make the preventative behaviour redundant. If Batfink is to avoid endangering human life, then it seems that a shut-down mechanism is indeed needed. Yet if it is not really the case that Batfink has the disposition to electrocute, why should the battery have to be disconnected in order to prevent living beings from frazzling? Unfortunately, however, there is an obvious move for the denier of IF dispositions to make. They can accept that the disconnection of the battery upon contact is necessary for the protection of life, but maintain that this is not because Batfink has the disposition to electrocute. Rather, it is necessary because if the modulator were not present, then Batfink would *gain* the disposition to electrocute. That is, were the features responsible for the intervention to be removed, a new disposition would come into existence, which explains why the preventative behaviour is necessary.

It is at this point, however, that the problems start for the deniers of IF dispositions. This is because they must then explain which of the features gained by Batfink during the modulator’s removal are the ones that plausibly constitute, or at least partially constitute (in conjunction, perhaps, with properties Batfink already has), the new disposition to electrocute. This is an extremely important issue, because if none of the features that Batfink already had before the modulator’s removal are ones that gave Batfink the disposition to electrocute, *then it had better be the case that by removing the modulator*, *Batfink is given a new property or properties of the sort that can plausibly constitute*, *or at least partially constitute*, *the disposition to electrocute.* If there are no such properties, then the existence of the new electrocution disposition would look mysterious. In that case, it would surely be better to say that the disposition was there all along, but that it was being finked.

The problem for the opponent of IF dispositions is that Batfink may not undergo any positive changes upon the removal of the modulator. Let us stipulate that Batfink’s modulator is easily detachable and so can be physically removed from Batfink without otherwise affecting its internal constitution or essential functions. In that case, what new properties are there that could explain Batfink’s new disposition to electrocute? If Batfink gains no positive properties upon the modulator’s removal, then there are only two possible kinds of property that Batfink could be said to gain: a new *negative* property or a new *totality* property, as will now be explained.

Let us begin by considering the state of play if all Batfink can be said to gain is a purely negative property, namely, the property of lacking a charge modulator. Negative properties are to be contrasted with positive properties because, unlike positive properties, negative properties concern only features that are *absent* from a thing. Is it plausible that negative properties could plausibly constitute, or at least partially constitute, an entirely new disposition?

Unfortunately for opponents of IF dispositions, it seems not, for as Armstrong (2004, p. 55), Molnar (2000, pp.77-9) and others have argued, it is hard to see how negative properties could constitute the causal grounds of dispositions. Dispositions, to recall, are taken by the participants in the fink debates to be genuine features of things. They are either taken to be irreducible entities in their own right or on more deflationist views they are identified with a categorical base. But on either view, dispositions are grounded in the concrete, spatiotemporally instantiated properties of things, properties which make a causal impact on the world. Negative properties, in contrast, concern what is *not* spatiotemporally instantiated. But for this reason, they are unsuitable candidates for conferrers of dispositions. A view which sees absences as somehow dictating how certain concrete things are disposed to behave looks very mysterious.

It should be conceded that we do sometimes appeal to absences when giving loose, everyday explanations for behavioural tendencies. For example, Molnar, who puts forward the sort of argument just outlined, concedes that not all causal explanations concern what is *operative* (Molnar 2003, pp. 77–9). When providing causal explanations, it is appropriate in certain contexts to contrast the explanandum with certain other events, and in constructing these contrastive explanations it can be natural to appeal to facts about absences. But if what we require is an account of what, *strictly speaking*, causally grounds the disposition to electrocute, as we currently do, then the ‘absence of modulator’ explanation is not sufficient. For, as we saw above, surely it is only the positive features of things that are able to do the world’s causal work.^3^

In short, if we take it that the only difference arising in Batfink upon the modulator’s removal is the acquisition of a negative property, then we are unable to identify a plausible source for the new disposition to electrocute. In that case, it is surely better to say that the disposition was there all along, conferred by the parts of Batfink we would ordinarily associate with that disposition, such as the battery supply.

This is not quite the end of the matter, however, because it is not wholly clear that Batfink would acquire only the purely negative property of lacking a charge modulator. It could be argued that once the charge modulator is removed, what Batfink gains is, to be precise, a new *overall* mass, for example, and a new *overall* internal structure. Although, after the alteration, we are not left with any internal structure that was not there before, it could be argued that Batfink has a new *total* structure, given that the former total structure included the modulator.^4^ States of affairs like these are cases that Armstrong (2004, pp. 61-2) calls *totality* or *limiting s*tates of affairs. They are limiting in that they involve a thing having properties to a certain degree *and nothing more*.^5^ One might notice, however, that the limiting ‘*and nothing more*’ clause has a distinctly negative flavour, which might make us wonder whether totality properties are just negative properties by another name (see e.g. Armstrong 1997, pp.200–201, who remarks that totality states of affairs clearly involve a relation to what is *not*). But as mentioned above, the overall-internal-structure property is at least partially about the positive internal structure that Batfink has, and so totality features seem not to be wholly negative, even though they do have a negative aspect to them.

Unfortunately for the deniers of IF dispositions, however, it is hard to see how the totality properties that remain upon the removal of the modulator *in the Batfink case* are of a sort that could plausibly confer a *new* disposition which was not already there. Crucially, given that the positive components of the relevant totality facts (concerning Batfink’s internal structure, say) were already present before the modulator’s removal, and given that it is denied that the disposition to electrocute is conferred until the removal of the modulator, *then it will have to be the negative aspect of the totality fact which serves to confer*, *at least in part*, *the new disposition to electrocute*. But as we saw earlier, a negative feature cannot plausibly endow a thing with new dispositions, even partially, given that they are concerned only with what is *not* spatiotemporally instantiated in the thing. In short, in order for new totality features to confer new dispositions, it would have to be the case that the relevant totality properties involve at least *some new positive features* which were not there before. But this is not so in the Batfink case, given the way it has been set up. At this point, then, a much neater story presents itself, which is that Batfink’s disposition to electrocute was there all along, conferred by the parts of Batfink we would ordinarily associate with such a disposition, such as the battery supply.

Now, if all of the above insights are generalized, we are clearly led to a new way of testing for the presence of an IF disposition. This criterion involves two conditions, the joint satisfaction of which is sufficient to show there is an IF disposition in a given case. The conditions are:

- *IF CONDITION 1*: *The object has a part which we would ordinarily think confers the disposition in question.*
- *IF CONDITION 2*: *The alleged intrinsic fink can be physically removed without otherwise making any positive alterations to the object in question.* 6

With this new criterion in play, let us finally consider how they deal with the everyday problem cases Choi presents for proponents of IF dispositions.

## Choi’s Counterexamples: Birds, Aluminium and Bricks

As indicated earlier, Choi argues that the standard reasons for accepting IF dispositions will lead us to ascribe strange dispositions in certain everyday cases. That they are everyday cases is important, for as Choi points out (Choi 2012, p. 303), we often do not have very clear intuitions about the dispositions involved in artificial cases, such as Clarke’s case of finkable human strength (Clarke 2008, p. 513) or the case of the shy chameleon (Clarke 2008, p. 513). Before showing how the new criterion proposed can deal with Choi’s counterexamples, it should also be noted that, as Choi highlights (Choi 2012, p. 306), strictly speaking his counterexamples involve masks rather than finks.^7^ But given that proponents of IF dispositions invariably accept intrinsically maskable dispositions for the same reasons as IF dispositions, Choi’s counterexamples still serve their intended purpose.

### The Flying Bird

Before examining the two cases which I agree create problems for Clarke and Fara, let us first set aside a case which I think is intuitively less compelling. The case in question involves a flying bird weighing *n* pounds. Given that, ordinarily, an object weighing *n* pounds would be disposed to fall down (when, say, released from a certain height), this suggests that by the reasoning Choi attributes to Clarke, Clarke should say that the flying bird also has the disposition to fall down, but that the wings act as an intrinsic antidote to that disposition. But according to Choi, ‘there is no doubt that the bird is not disposed to fall down’ (Choi 2012, p. 308). If Choi’s intuition is correct, then this would clearly be a problem for both Clarke and the account of IF dispositions offered above. Regarding the latter, one could surely remove the bird’s wings without otherwise altering its positive properties, in which case IF condition 2 (as well as condition 1) is clearly satisfied. In response, however, I can only report that Choi’s assessment in this particular case strikes me as being straightforwardly mistaken, and that intuitively the bird does indeed have the disposition to fall down. I would urge Choi to reflect on the fact that, since the flying bird has to constantly work hard to keep itself in the air, it is surely proper to say that the bird is disposed to fall down in some sense.^8^ Of course, we might change our assessment of the case for theoretical reasons, but if we are just going by intuition, as Choi is, then this case is far less compelling than the two problem cases now to be discussed.^9^

### Aluminium

Let us now move on to a better example from Choi. Is aluminium disposed to rust, Choi asks (Choi 2012, p. 306)? Intuitively, it is not, according to Choi, because when exposed to oxygen, aluminium produces a dense skin of aluminium oxide which immediately protects it from the levels of oxidation that would be necessary for it to form a reddish-brown residue (i.e. rust). However, because aluminium has the same kind of microstructure as metals which clearly do have the disposition to rust, it seems, prima facie, that proponents of IF dispositions like Clarke should conclude that aluminium does have the disposition to rust. I agree with Choi’s intuition that aluminium does not have the disposition to rust. But the analysis proposed earlier is able to deliver the correct verdict in this case. Recall that on this analysis, IF condition 2 tells us that it is important that the putative intrinsic fink can be physically removed without otherwise making any positive alterations to the thing in question. But this condition cannot plausibly be satisfied in the aluminium case, and so we can agree that it does not have the disposition to rust. The problem is that aluminium, *by its very nature* will always create a think protective film when oxidised. If some substance did not create a film of aluminium oxide upon oxidation, then that substance would not be aluminium. What is the relevance if this? Well, it means that the only way in which we can eliminate aluminium’s protective behaviour is to transform it into some other substance. And that will inevitably leave us with something that has new positive chemical properties and internal structure. For instance, we could eliminate aluminium’s protective behaviour by creating an alloy from it. It is well known that aluminium alloy, as opposed to pure aluminium, offers little protection against corrosion. But aluminium alloy has very different positive properties from pure aluminium. For instance, although copper and aluminium are both relatively soft metals, an alloy combining the two is much harder and stronger.

Before moving on, it is important to note that it would perhaps be harmless enough to say that a lump of aluminium has the capability to *gain* the disposition rust, were certain positive, substantial changes to be brought about. What this suggests is that we should be careful to distinguish the notion of an intrinsically finkable or maskable disposition from the notion once introduced by Shoemaker of a *conditional* power. Shoemaker says, for example, that a knife-shaped piece of butter has the power to cut wood conditionally upon being turned to steel, i.e., it has the conditional power to cut wood (Shoemaker 2003, p. 212). In other words, the butter has the potential to gain the ability to cut wood, given certain positive changes. The aluminium case is clearly more analogous to this case than cases involving IF dispositions. Like aluminium, the butter would have to undergo a positive change in order to gain the power to cut, for instance by being being deep frozen or, to use Shoemaker’s own example, by (somehow) becoming steel.

Importantly, this observation may help to explain why there has been hostility in some quarters towards the idea of IF dispositions. The reason is that many of the cases that a proponent of IF dispositions might initially suppose involve IF dispositions are in fact more like cases of Shoemaker’s conditional powers, thereby creating confusion on both sides. But I will not be able to explore this diagnosis further here.

### The Brick

The final example to be discussed is one that Choi attributes to Fara’s work (Fara 2008, p. 857). Does a brick have the disposition to roll down a hill, despite the fact that it is rectangular? Clearly, the answer is ‘no’, yet one could perhaps conceive of a rectangular brick as a spherical object whose disposition to roll is frustrated by the outer parts of the brick (i.e. the parts that give it an overall rectangular shape). The danger facing proponents of IF dispositions, then, is that they will have to accept that a rectangular brick has the disposition to roll, which is absurd. In response, Fara (2008, p. 857) argues that this case does not involve the disposition to roll because the putative masking factor is a permanent feature of the brick. According to Fara’s analysis, we should only ascribe intrinsically finkable/maskable dispositions in cases where the finking/masking factors are temporary features of the things in question. But despite this account’s initial promise, Choi argues that Fara’s criterion breaks down in modified cases. Suppose, Choi says, that a brick regularly changes its shape—from being, say, rectangular to being spherical (Choi 2012, p. 310). In such a case the overall shape of the brick is a temporary rather than permanent feature of the brick and yet we still would not want to say that, during the rectangular periods, the brick has the disposition to roll.

Fortunately, however, the new criterion introduced earlier does arguably allow us to deny that a rectangular brick has the disposition to roll. It should be conceded that IF condition 2 is plausibly satisfied because the rectangular-making parts of the brick could be physically removed (using a chisel, say) without otherwise bringing about any positive changes. But recall IF condition 1, which requires that the thing has a part which we would ordinarily think confers the disposition in question. Does the brick really have a spherical part? I find it very strange to think that a rectangular brick has a spherical part. At least, not if we are using our everyday notion of ‘part’.^10^ If we are to allow that the brick has a spherical part, then surely we could arbitrarily pick any of an infinite number of shapes and say that it has a part with that shape. Again, I take it that this would be a very unnatural thing to say. Perhaps we can conceive *in thought* of many inner overlapping areas of the brick having a certain shape, by abstracting the rest of the brick away. But these are purely divisions that are being created and projected in the mind. It would be strange indeed to say that the homogenous brick really is divided up in that way. I take it, for instance, that we would all agree that while (for example) there is a definite sense in which a garden wall has brick-shaped parts, we would not want to say that it also has spherical parts, star-shaped parts, dog-shaped parts, butterfly-shaped parts, and any other arbitrarily shaped part we care to mention.

## Conclusions

In this paper we have seen that Choi’s examples create a significant problem for existing accounts of IF dispositions. I have, however, provided a new argument for the existence of IF dispositions, one which delivers a new way of testing for the presence of such dispositions With this new account in place, proponents of IF dispositions can deliver intuitively correct verdicts in Choi’s examples. So, it is not time, yet, to give up on the possibility of intrinsically finkable or maskable dispositions.

## Footnotes

1 Choi (2010, p. 585) also relies on his rejection of intrinsic finks in his response to Lee (2010), who criticizes Choi’s simple conditional analysis of dispositions.

2 After this paper was submitted for review at this journal, a paper by Kittle (2015) appeared elsewhere which has similar broad aims. That is, Kittle responds to critics of intrinsically finkable dispositions like Choi by trying to draw a principled distinction between cases which do involve genuine intrinsically finkable dispositions and those which do not. However, Kittle’s strategy is very different to mine, for it involves appealing to considerations about the role of ‘circumstances’ surrounding the stimulus and manifestation of a putative disposition (2015, p. 374) and also the ‘modal strength’ of dispositions (2015, p. 379). Clearly, if Kittle’s arguments succeed in strengthening further the case for intrinsically finkable dispositions, then I would welcome this.

3 If there is any remaining doubt that it is only the positive properties of the world which are causally potent, we may also allude to the fact that the physical causal laws in science invariably involve properties which are wholly positive in nature. In fact, Armstrong takes this to be a good reason for naturalists like himself to deny that negative properties (or what he calls negative ‘universals’) are genuine natural properties at all.

4 Handfield (2008) is one who I think would appeal to changes in totality properties in order to explain the alleged appearance of the new disposition. In one place, for instance, he suggests that dispositions are determined by the ‘total complement of supervenience base properties’ (2008, p. 302).

5 Compare Batfink’s having a certain internal structure *and nothing more* with the state of affairs, discussed by Armstrong (2004, pp. 61-2), of a methane molecule having a structure involving five atoms *and that is all.*

6 If the alledged fink satisfies this condition, then it would be classed as a ‘destructive interferer’ as defined by Contessa (2013, p. 407). Given that Contessa’s analysis of dispositions classifies cases of destructive interference as genuine cases of finkishness, the arguments of this paper lend support to the sort of analysis of dispositions he offers as opposed to that offered by Choi (2012, p. 291).

7 In masking cases, the putative disposition is not removed when the triggering circumstances occur as they do in finkish cases, but nonetheless the expected manifestation is blocked by the masking factor (the ‘antidote’).

8 This means that, intuitively, the bird has opposing dispositions: the disposition to fly and the disposition to fall down (though of course only one of these dispositions can be manifested at a given time). But I agree with Clarke that there is nothing problematic about things having opposing dispositions. See Clarke 2010.

9 Incidentally, I would say the same about the lactose case which is discussed by Handfield and Bird (2008), and which Choi briefly alludes to (2012, p. 308). According to Handfield and Bird (2008, p. 291) it would be absurd to say that most people have the disposition to be poisoned by milk when ingested, given that lactase is usually present in their bodies. However, as with the bird case, it is less clear to me that it would be absurd to ascribe such a disposition in this case. Furthermore, even if we agree with Handfield and Bird that the presence of lactase in a person is an intrinsic feature of the person (which may itself be questionable), surely the lactase is an extrinsic antidote relative to the milk that is ingested. Hence, if we allow dispositions which can be extrinsically finked/antidoted, Handfield and Bird’s verdict seems to leave us with an odd asymmetry: the milk is disposed to poison the person when ingested but that same person is not disposed to be poisoned by the milk when ingested.

10 I do not doubt that some metaphysical notion of part can be stipulated on which it is true to say that the brick has a spherical part. But such a notion is far removed from the ordinary notion of a part in play in what are our intuitive assessments of these cases.
